# Artificial Neurons Based on Ag/V_2_C/W Threshold Switching Memristors

**DOI:** 10.3390/nano11112860

**Published:** 2021-10-27

**Authors:** Yu Wang, Xintong Chen, Daqi Shen, Miaocheng Zhang, Xi Chen, Xingyu Chen, Weijing Shao, Hong Gu, Jianguang Xu, Ertao Hu, Lei Wang, Rongqing Xu, Yi Tong

**Affiliations:** 1 College of Electronic and Optical Engineering & College of Microelectronics, Nanjing University of Posts and Telecommunications, Nanjing 210023, China; 2020020114@njupt.edu.cn (Y.W.); 1219023530@njupt.edu.cn (X.C.); b18020308@njupt.edu.cn (D.S.); zmcstudy@163.com (M.Z.); 1320027503@njupt.edu.cn (X.C.); iamethu@njupt.edu.cn (E.H.); leiwang1980@njupt.edu.cn (L.W.); 2 Gusu Laboratory of Materials, Suzhou 215000, China; shaoweijing2020@gusulab.ac.cn (W.S.); guhong2021@gusulab.ac.cn (H.G.); 3 Institute of Advanced Materials (IAM), Nanjing University of Posts and Telecommunications, Nanjing 210023, China; 1220066008@njupt.edu.cn; 4 School of Materials Science and Engineering, Yancheng Institute of Technology, Yancheng 224051, China; jgxu@163.com

**Keywords:** MXene, memristor, threshold switching, leaky integrate-and-fire, artificial neuron

## Abstract

Artificial synapses and neurons are two critical, fundamental bricks for constructing hardware neural networks. Owing to its high-density integration, outstanding nonlinearity, and modulated plasticity, memristors have attracted emerging attention on emulating biological synapses and neurons. However, fabricating a low-power and robust memristor-based artificial neuron without extra electrical components is still a challenge for brain-inspired systems. In this work, we demonstrate a single two-dimensional (2D) MXene(V_2_C)-based threshold switching (TS) memristor to emulate a leaky integrate-and-fire (LIF) neuron without auxiliary circuits, originating from the Ag diffusion-based filamentary mechanism. Moreover, our V_2_C-based artificial neurons faithfully achieve multiple neural functions including leaky integration, threshold-driven fire, self-relaxation, and linear strength-modulated spike frequency characteristics. This work demonstrates that three-atom-type MXene (e.g., V_2_C) memristors may provide an efficient method to construct the hardware neuromorphic computing systems.

## 1. Introduction

In the big data era, the traditional Von Neumann architecture is facing challenges such as the memory wall [1,2]. The separation of memory and computing units brings more high-power and expensive costs in the existing complementary metal oxide semiconductor (CMOS) circuitry systems. To remove this bottleneck, neuromorphic computing based on spiking neural network (SNN) has emerged as an efficient solution to realize a more efficient computing system [3,4]. Principally, SNN performs a computational task with asynchronous and sparse spikes which enable a high similarity to the human brain on artificial neuromorphic hardware due to its noise resiliency, energy efficiency, and convenient implementation of spatiotemporal learning rules [2]. Consequently, SNNs are promising for more faithful and efficient neuromorphic computing systems. From the perspective of hardware implementation, it is mandatory to explore artificial synapse and neuron for neuromorphic systems [5,6]. Memristor, known as the fourth basic circuit element, has been investigated to implement artificial synapses and neurons due to its nanoscale, non-volatile memorability, and nonlinearity characteristics [7,8,9,10,11]. To date, reversible artificial synapses have been reported using resistive switching memristors [12,13,14,15,16]. In contrast, investigation on realizing an artificial neuron based on a single device is not well-explored. Recently, some emerging materials (i.e., transition metal oxides, phase-change materials, organic–inorganic halide perovskites, and 2D materials) have been utilized to realize the artificial neuron based on a single memristor, which may bring new insight to this field [17,18,19,20,21,22,23,24].

Two-dimensional (2D) van der Waals heterostructures have provided new feasibilities to fabricate neuromorphic nano devices due to their excellent structural stability and various physicochemical properties [11,25,26,27,28]. Among them, MXene, a family of two-dimensional (2D) transition-metal carbides, carbonitrides, and nitrides, has shown interesting semiconductor characteristics owing to their abundant electrochemically active surfaces [29,30,31,32]. For example, previous reports have been investigated on the MXene (Ti_3_C_2_)-based memristors to realize fast pulse modulation time and emulation of neuromorphic behaviors [20,33,34,35,36]. In particular, it is reported that three-atom-type MXene (e.g., V_2_C) exhibited ultra-low power, more stable endurance, and multiple synaptic functions, i.e., short-term plasticity (STP), long-term plasticity (LTP), spike-timing-dependent plasticity (STDP), and spike-rate-dependent plasticity (SRDP), due to its more stable atomic structure and higher conductivity [37,38,39]. Notably, to date, there are few reports on emulating an artificial neuron by three-atom-type MXene-based memristors. Both the artificial neuron and the synapse are two essential elements for constructing SNNs [40]. Therefore, it is imperative to develop a kind of MXene-based device suitable for efficient neurons.

In this work, we report a leaky integrate-and-fire (LIF) artificial neuron based on a single memristor made of V_2_C MXene materials (Ag/V_2_C/W), which exhibits repeatable threshold switching characteristics under a low compliance current (I_CC_) of 0.10 μA. This volatile behavior can be explained by diffusive Ag ions in the V_2_C layer. Moreover, multiple neural features including leaky integration, threshold-driven fire, and self-relaxation have been faithfully emulated via a single V_2_C memristor without auxiliary circuits. Furthermore, the relationship between necessary integration time and input spiking frequency has been explored, which well mimics the strength-modulated spike frequency characteristics of biological neurons. Finally, the increasing amplitude of input pulses leads to increasing fire frequency based on remarkable linear fitting, which demonstrates the possibility of a V_2_C-based artificial neuron for the application of neuromorphic computing.

## 2. Materials and Methods

Vanadium carbide (V_2_C) MXene powders were obtained from the precursor V_2_AlC by strong acid etching. Furthermore, after mixing 2 g lithium fluoride (LiF) and V_2_AlC powders in 40 mL hydrochloric (HCl) acid, the mixture was stirred for 72 h at 90 °C. The obtained suspension was then washed until the pH was neutral, using deionized water. Finally, the V_2_C powders were collected through centrifuging, physical evaporation, and vacuum-drying. Particularly, the surface morphology of V_2_C was investigated by scanning electron microscope (SEM) (HITACHI Ltd., Tokyo, Japan). The X-ray diffraction (XRD) pattern (Malvern Panalytical B.V., Almelo, Netherlands) and X-ray photoelectron spectroscopy (XPS) (Thermo Fisher Scientific Inc., Waltham, MA, USA) have been used to confirm the composition of V_2_C MXene in this work. For the preparation of the V_2_C layer of the device, V_2_C powders were distributed in the deionized water to obtain the suspension for spinning coating later.

From bottom to top, the proposed Ag/V_2_C/W memristors were fabricated as follows. First, the metallic tungsten bottom electrode of 80 nm was deposited on a 300-μm-thick SiO_2_/Si wafer by magnetron sputtering. Next, the prepared V_2_C suspension was deposited onto the W electrode by spin-coating at 1500 rpm for one minute. The remaining liquid was removed by evaporation at 90 degrees centigrade for 20 min to deposit a uniform V_2_C film, which serves as the active layer of memristors. Finally, a 100 nm Ag top electrode was sputtered onto the V_2_C film with a square size of 500 × 500 μm^2^. In addition, the cross-sectional image of these V_2_C memristors has been characterized by SEM. Furthermore, the electrical characteristics were measured by Keithley 4200A-SCS Semiconductor Characterization Analyzer (Tektronix Inc., Beaverton, OR, USA).

## 3. Results and Discussion

**Figure 1:**
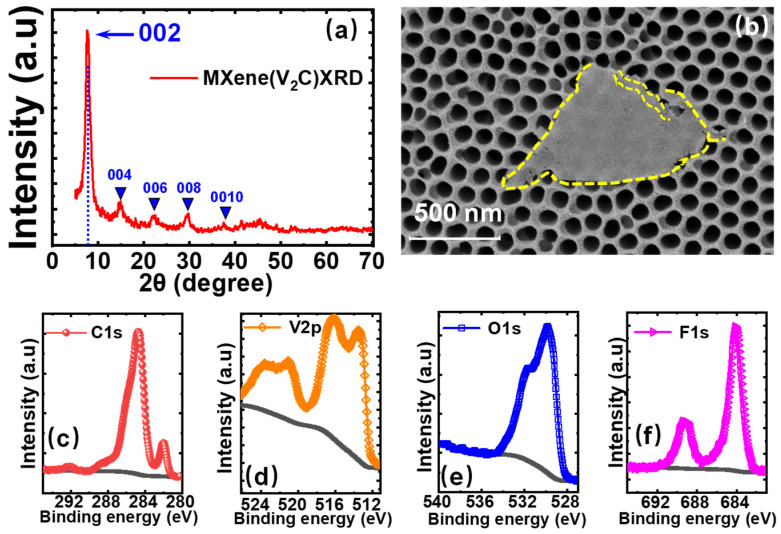
Material characterization of V_2_C MXene. (**a**) XRD pattern of V_2_C powders used in this work. (**b**) SEM image of prepared V_2_C powders on the substrate of porous alumina. (**c**–**f**) XPS spectra of V_2_C MXene for (**c**) V2p (**d**) C1s (**e**) O1s, and (**f**) F1s.

Figure 1a shows the XRD pattern of V_2_C powders based on the glass substrate, which exhibits strong peaks at ~8.0 degrees (002). Other weak peaks appear at 15.0 degrees (004), 22.1 degrees (006), 29.7 degrees (008), and 37.5 degrees (0010), corresponding to different planes of newly formed V_2_C MXene [41,42]. After the synthesis of V_2_C, the V_2_C solution was dropped on the porous alumina to characterize the surficial morphology by SEM. As shown in Figure 1b, few layered V_2_C nanosheets can be observed clearly. Additionally, the elemental compositions of V_2_C were explored by XPS. The XPS spectra for V, C, F, and O of this MXene V_2_C sample are plotted in Figure 1c–f, respectively.

**Figure 2:**
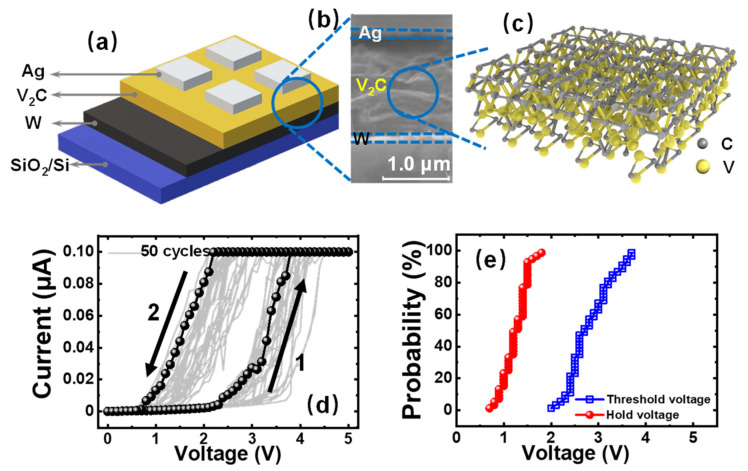
Schematic and DC electrical performances of V_2_C-based memristors. (**a**) Schematic illustration of the proposed Ag/V_2_C/W memristors. (**b**) Cross-sectional SEM image of the fabricated memristor. (**c**) Atomic structure of few layered V_2_C MXene. (**d**) Fifty sequential cycles of I-V characteristics of V_2_C memristor with linear scale. (**e**) Distribution of threshold and hold voltage extracted from Figure 2d.

After the fabrication of the device, Figure 2a illustrates the structure of Ag/V_2_C/W memristors. Furthermore, the cross-sectional SEM image clearly shows the three layers of the device, as shown in Figure 2b. It can be indicated that the thickness of the V_2_C layer inserted in our memristors is about 1.5 μm. Additionally, the atomic structure was constructed according to the single V_2_C unit with the pristine structure of a hexagon, including two vanadium atoms (yellow balls) and one carbon atom (grey balls) in Figure 2c.

To investigate the electrical characteristics of V_2_C memristors, a direct current (DC) sweeping voltage of 5.0 V was applied on the device. Figure 2d demonstrates the 50 sequential cycles of typical threshold switching I-V curves under an I_CC_ of 0.10 μA. As the sweeping volage arrives at threshold voltage (V_th_), the resistance was switched from OFF to ON state. Then this device was automatically switched back to OFF state at hold voltage (V_hold_). Additionally, to investigate the operating voltage of the TS behavior, the distribution of V_th_ and V_hold_ has been plotted in Figure 2e. It can be observed that V_hold_ (~1.2 V) is more stable than V_th_ (~3.1 V) of the Ag/V_2_C/W memristor. Still, there is a window between V_th_ and V_hold_, which shows potential for neuromorphic circuits design [43,44].

**Figure 3:**
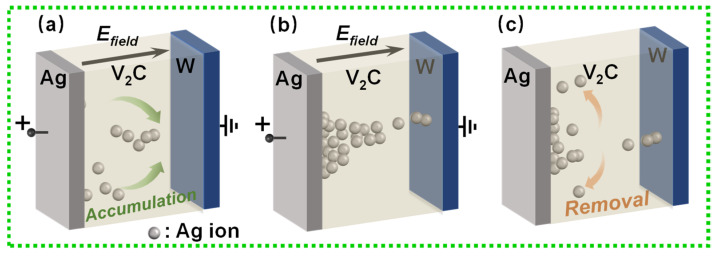
Illustration of dynamic conductive silver (Ag) ions in V_2_C-based memristors. (**a**) Diffusive process of Ag ions in TS mechanism. (**b**) ON state constructed by Ag conductive filaments. (**c**) Automatic removal of conductive filaments without bias.

Figure 3 shows the schematic of conductive filaments (CFs) models. As shown in Figure 3a, Ag ions were initially produced under the positive electrical stimulation. Then, Ag ions initiate the accumulation of silver CFs, shown in Figure 2b with the structure of semi-finished CFs. However, when the applied voltage was not strong enough, filaments were spontaneously ruptured resulting from the Joule heat effect and minimum energy positions [45,46]. Furthermore, our previous work has presented the transition from volatile to non-volatile switching realized by increasing I_CC_ in the SET process [37]. In conclusion, this unique non-nonlinearity of the TS mechanism can be applied to act as a potential for selectors of large-scale RRAM arrays and a biological emulator of neural behaviors [46,47].

**Figure 4:**
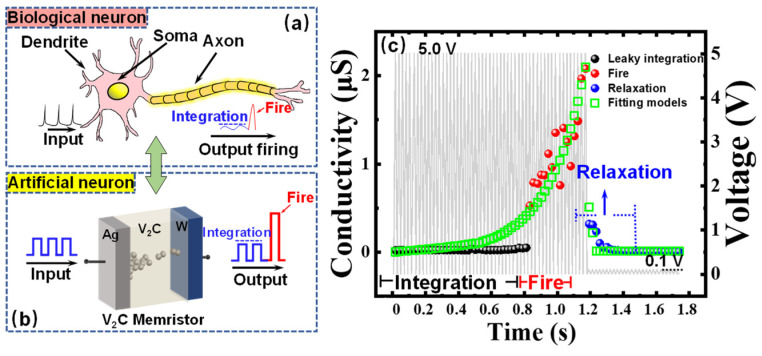
Experimental implementation and fitting model of a leaky integrate-and-fire (LIF) neuron realized by a single V_2_C-based memristor. (**a**) Schematic of a biological neuron receiving input from other neurons through interconnected synapses. (**b**) Diagrammatic illustration of an artificial neuron emulated by an independent V_2_C memristor. (**c**) Plot of response conductivity and fitting models (green squares) of the integration (black balls), fire (red balls), and relaxation (blue balls) function of the device under a train of input voltage pulses (grey).

In a biological neuron, the input spikes from other neurons are transited to this output neuron, which boosts the membrane potential (MP). Meanwhile, the MP will leak out until it reaches a threshold value. After exceeding this threshold, the neuron will trigger the spikes into the axon, known as output firing, as plotted in Figure 4a [48,49]. Accordingly, Figure 4b illustrates the experimental realization of a LIF neuron based on a single V_2_C memristor. In this work, one single V_2_C-based memristor was in terms of controlling the emulation of the LIF neuron accurately. Thanks to the dynamic transitions of Ag ions in V_2_C layer, the conductivity of memristor could faithfully mimic the MP in a biological neuron. Under a strain of input pulses with an amplitude of 5.0 V, width of 11 ms, and frequency of 48 Hz, the V_2_C-based memristor electrically emulated the functions of leaky integration, fire, and relaxation in LIF neurons. It can be observed from Figure 4c that the conductivity increased sharply at a value of ~0.5 μS under this train of input pulses. Furthermore, the firing conductivity raised at ~2.1 μS. The variation of conductivity at the earlier stage of firing process may be attributed to the weak Ag conductive filaments. To investigate the neural function of relaxation, the input signals were replaced by reading pulses with an amplitude of 0.1 V. The period of relaxation was measured about 0.30 s from the plot. The corresponding differential equation of LIF model is:

(1) $$C_{m}\frac{dV}{dt}=I_{app}-G_{L}\left(V-E_{L}\right)$$

$C_{m}$ represents the capacitance of the cell membrane surface, $I_{app}$ represents the input current, $G_{L}$ is the conduction of the leak model, $E_{L}$ is the passively balanced voltage [50,51].

When $t_{0}=0$, assuming $V\left(t_{0}\right)=0$, if the input current is a series of short pulses ($\Delta \;\ll \frac{C_{m}}{G_{L}}$) (as the set up in the experiment), carrying out a Taylor series expansion of the membrane potential, it can be found that at the end of the pulse, the membrane potential is only related to the total charge flowing through, as follows:

(2) $$V\left(\Delta \right)=\frac{I_{0}\Delta }{C}=\frac{q}{C}$$

As shown in Figure 4c, these points on the models (green squares) fit well with the experimental data of LIF neuron. The emulation of a LIF neuron has been experimentally emulated by a single V_2_C-based memristor without any auxiliary circuits, which may provide a low-cost candidate for the implementation of artificial neurons in a neuromorphic system.

**Figure 5:**
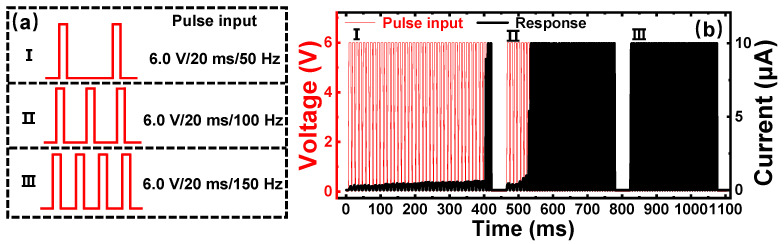
Current response of different frequency of input pulses. (**a**) Trains of pulses with different frequency of 50 Hz (first), 100 Hz (second), and 150 Hz (third). (**b**) Current response of sequential input pulses corresponding to (**a**).

To further explore the frequency characteristics of V_2_C-based neurons, different programming input pulses have been attempted on our devices. As illustrated in Figure 5a, the input electrical force was divided into three groups as follows: first, 6.0 V (amplitude)/20 ms (width)/50 Hz (frequency); second, 6.0 V/20 ms/100 Hz; and third, 6.0 V/20 ms/150 Hz. The current response has been illustrated in Figure 5b. Red curves illustrate the input pulses and black curves illustrate the response current. From the electrical curves of the V_2_C-based neuron, the integrate and fire neural functions have been observed under the first train of pulses. Additionally, the first group demonstrates that the necessary integration time to trigger the fire is ~0.39 s. Correspondingly, for the second group, the necessary integration time of ~0.06 s was shortened from the previous test. Furthermore, the device only needs one pulse (~0.02 s) to achieve the conductivity threshold resulting in the behavior of fire under the input of the third group. Consequently, the response results indicate that the frequency of input is positively associated with the necessary integration time of memristors. For this reason, it is believed that inputs with fast frequency can promote the fast growth of Ag CFs in the V_2_C, reducing the time of the integration process. In perspective, this property could be used to investigate the appropriate frequency of input signals in order to cut the power consumption for units of artificial neurons [52].

**Figure 6:**
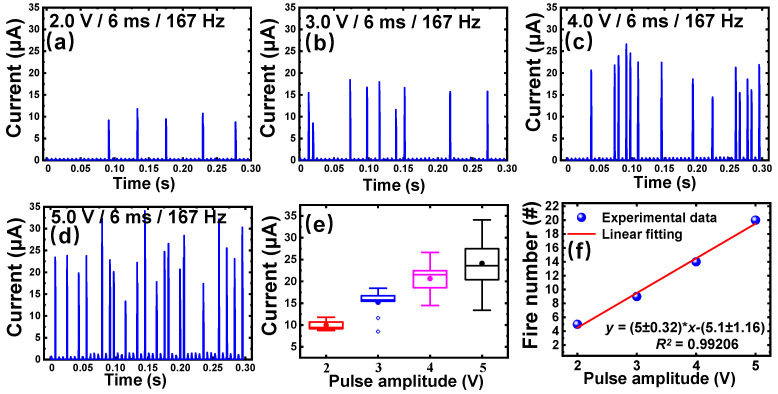
Output results of firing frequency influenced by the amplitude of the applied pulses. (**a**–**d**) Responses of firing frequency to impulse sequences with amplitude/width/frequency of (2.0 V/6 ms/167 Hz), (3.0 V/6 ms/167 Hz), (4.0 V/6 ms/167 Hz), and (5.0 V/6 ms/167 Hz), respectively. (**e**) Box chart of current response to input pulses with different amplitudes. (**f**) Plot and linear fitting of firing frequency related to programing input pulses with different amplitudes extracted from (**a**–**d**).

Biologically, the firing frequency increases with increased stimulus strength, called the strength-modulated spike frequency characteristic [53]. As shown in Figure 6a,b, a series of input pulses (6 ms width, 167 Hz frequency) with different amplitudes (2.0 V, 3.0 V, 4.0 V, and 5.0 V) has been applied to V_2_C-based memristors, respectively. It should be noted from the plots that firing currents also increase with increased pulse amplitudes. The electrical variation of firing spikes can be explained by the ionic process of Ag particles detaching from a Ag reservoir [54,55]. Additionally, the firing frequency clearly increases with increasing input pulse amplitude.

To statistically demonstrate these properties, the box chart in Figure 6e exhibits the statistical results of the firing current with stimulus strength. As plotted in Figure 6f, the input pulse amplitudes increased with amplitudes of input, which is a linear relationship well fitted by y = (5 ± 0.32) · x − (5.1 ± 1.16), wherein the R-square is of ~99.206%.

To explore these mechanisms, the dynamic voltage-driven ion movement can be used to explain the strength-modulated spike frequency characteristic in our devices. Under low strength stimulation, the voltage-driven ions are easier to diffuse back to the original position due to the existence of a built-in electric field, leading to the small electrical response. On the other hand, as input pulses with high amplitudes are applied on the device, ions are harder to diffuse back to the initial position, resulting in the current enhancement effect and high firing frequency. We assume that the higher diffusion coefficient of Ag ions in V_2_C may enhance the diffusive process of Ag for threshold switching and emulate faster and more controllable neurons [45,56,57]. In conclusion, the superior strength-modulated spike frequency characteristic has been successfully implemented, which may strengthen the feasibility of MXene-based artificial neurons for neuromorphic systems [18,58,59,60].

## 4. Conclusions

In conclusion, Ag/V_2_C/W memristors have been fabricated, exhibiting threshold switching characteristics under a low I_CC_ of 0.10 μA. The diffusive Ag ions could explain this non-volatile switching mechanism. Then, the as-designed V_2_C-based memristors have faithfully emulated the biological neurons without auxiliary circuits, including leaky integration, threshold-driven fire, and self-relaxation neural functions. In V_2_C-based neurons, leaky integration time, and firing frequency can be regularly modulated under the different strength of stimulus. Furthermore, the strength-modulated spike frequency characteristics have been achieved by a superior linear relation between input amplitudes and firing frequency of V_2_C-based memristors. Finally, this work may provide a simple candidate to construct efficient neuromorphic computing devices for SNNs. We will try to implement the hardware neural networks with V_2_C-based neurons in our future work.

## Data Availability

The data presented in this study are available on request from the corresponding author.
